# Ebola vaccine development plan: ethics, concerns and proposed measures

**DOI:** 10.1186/s12910-016-0094-4

**Published:** 2016-02-08

**Authors:** Morenike Oluwatoyin Folayan, Aminu Yakubu, Bridget Haire, Kristin Peterson

**Affiliations:** Institute of Public Health and Department of Child Dental Health, Obafemi Awolowo University, Ile-Ife, Nigeria; National Health Research Ethics Committee, Federal Ministry of Health, Federal Secretariat, Abuja, Nigeria; Kirby Institute for Infection in Society, UNSW, Australia; Department of Anthropology, University of California, Irvine, CA USA

**Keywords:** Ebola, Vaccine, Healthcare workers, TRIPS, Public dialogue, Standard of care, Myths

## Abstract

**Background:**

The global interest in developing therapies for Ebola infection management and its prevention is laudable. However the plan to conduct an emergency immunization program specifically for healthcare workers using experimental vaccines raises some ethical concerns. This paper shares perspectives on these concerns and suggests how some of them may best be addressed.

**Discussion:**

The recruitment of healthcare workers for Ebola vaccine research has challenges. It could result in coercion of initially dissenting healthcare workers to assist in the management of EVD infected persons due to mistaken beliefs that the vaccine offers protection. It could also affect equity and justice. For example, where people who are not skilled health care professionals but who provide care to patients infected with Ebola (such as in home care settings) are not prioritized for vaccination. The possibility of study participants contracting Ebola infection despite the use of experimental vaccine, and the standard of care they would receive, needs to be addressed clearly, transparently and formalized as part of the ethics review process. Future access to study products in view of current status of the TRIPS agreement needs to be addressed. Finally, broad stakeholder engagement at local, regional and international levels needs to be promoted using available communication channels to engage local, regional and international support. These same concerns are applicable for current and future epidemics.

**Summary:**

Successful Ebola vaccine development research requires concerted efforts at public dialogue to address misconceptions, equity and justice in participant selection, and honest discussions about risks, benefits and future access. Public dialogue about Ebola vaccine research plans is crucial and should be conducted by trusted locals and negotiated between communities, researchers and ethics committees in research study sites.

## Background

The recent outbreak of Ebola Virus Disease (EVD), which is still ongoing in Guinea and Sierra Leone, is the worst in history. The spread of the EVD from Africa to Europe and North America has heighted international concerns about EVD including the prospect for the virus being used for bioterrorism [1]. The recognition of thecatastrophic potential of EVD has also brought a sense of urgency to the development of therapeutic and preventative interventions. Many governments and donors are not only devoting financial and human resources to support the response to the current outbreak in West Africa, they are also supporting research for improved control of EVD [2].

At present, there is no approved specific therapy or vaccine for EVD. Supportive clinical care, which includes aggressive fluid and electrolyte replacement, and antibiotic therapy have been the most promising care for EVD patients in the affected West African countries [3]. The use of antiemetics and antidiarrheal medications is also important to reduce massive gastrointestinal losses and the consequences of hypovolemic shock [4–7]. Providing optimal intensive care services, including options like dialysis that are available in resource-rich countries [8, 9], are likely to save more lives, but are widely unavailable in affected resource-poor countries.

While new therapeutic approaches and new preventive interventions are often discussed together, it is important to distinguish between them when considering both the potential benefits and the ethical challenges involved in their research and development processes. The development of treatments that directly target the Ebola virus offer the greatest promise of reducing the case fatality associated with EVD, while the development of preventative vaccines would be valuable in reducing transmission and limiting the size of future epidemics.

Also, multiple reports allude to the weakened health systems in the region as a significant factor in the spread of EVD [10, 11]. Additionally, there are historical and cultural factors that limited the early control of the outbreak. The history of government mistrust [12], denial of the existence of EVD [13, 14] and the prevalent myths and misconceptions about EVD including conspiracy theories about Northern exploitation of the South. The latter include the real histories of the slave trade, European colonialism, unfavorable economic and trade policies, and resource conflicts, all of which involved Northern interventions in alliance with government and other national elites [15]. Conducting EVD vaccine research poses ethical challenges in a region that possesses low confidence in the goodwill of government and foreigners – a challenge that is all the more difficult given low scientific literacy as well as a limited time frame in which to conclude research before the epidemic ends.

One of the key issues under discussion with regard to testing EVD vaccines is finding the appropriate population group to participate in the research. Healthcare workers who are involved with case management are at high risk of contracting EVD because of their close contact with patients during the most infectious period: when patients are highly symptomatic [15]. They are therefore prioritized as study participants for EVD vaccine research [16].

This article specifically addresses concerns and contexts pertaining to EVD vaccine development. We present ethical arguments against plans to exclusively target professional healthcare workers for experimental EVD vaccineII/III clinical studies [17]. We suggest there are fairer ways to prioritize people at high risk of infection – which includes, but is not limited to professional healthcare workers – than giving only one group of people privileged access to experimental products based on occupational status. We will further discuss the ethical challenges pertaining to the design and implementation of EVD vaccine research. These concerns include the appropriateness of randomization of study participants to control arms and the adequacy of care for vaccine trial participants [18], issues that are subject to ongoing debate [19–25]. We will also argue for the critical need to create and sustain community engagement processes and to promote community-level understanding of EVD vaccines; these are especially important considering the real concerns about the level and nature of community engagement efforts in the field [26]. The issues of access to proven effective EVD vaccines by poor governments and or communities will also be addressed. Finally, we discuss the need to build human capacity for health research and programming purposes because prospects for future emerging diseases require prompt regional responses.

## Debate

### Current EVD vaccine candidates and associated ethical dilemmas

The preliminary result of the phase III *Ebola ça Suffit* trial, which tested the vaccine candidate rVSV-ZEBOV were released on 31 July 2015. The result showed promising evidence of high vaccine efficacy: no participants who received immediate vaccination developed EVD 10 days or more after the contact (10–21 days is the incubation period and so, developing EVD prior to 10 days indicates that the person may have had exposure prior to the vaccination) [27]. The trial used an adaptive design modelled on the ‘ring’ vaccine strategy, previously used to eradicate smallpox in the 1970s (though in the case of smallpox, a known effective vaccine was used) [28]. ‘Ring’ methodology uses a contact tracing approach to first identify people known to have been in contact with a diagnosed index case, and then to offer them vaccination. In order to have an experimental aspect in the *Ebola ça Suffit* trial, communities participating in the trial were treated as ‘clusters’, and randomized either to immediate vaccination of contacts upon identification of an index case, or delayed vaccination, with contacts vaccinated 21 days after contact with the index case.

A key feature of the *Ebola ça Suffit* design is that it did not have a placebo arm. Also, it prioritized access to the vaccine candidate for all people at highest risk (i.e., those who had had contact with a diagnosed index case). The randomization to immediate vaccination compared with delayed vaccination, using carefully calibrated timing, meets the principle of distributive justice while still allowing experimental comparison between groups.

The rVSV-ZEBOV vaccine candidate is also being tested in two other trials: a phase II study in Guinea that ran in parallel with the ‘ring’ study and targeted 1200 ‘frontline’ workers (healthcare workers and other personnel such as members of laboratory, ambulance, burial or surveillance teams) [29]; and a phase III study in Sierra Leone called the STRIVE trial [30]. A third study called the PREVAILstudy [31], was to test rVSV-ZEBOV against another candidate, ChAd3-EBO Z, and placebo, in a general population group. This third trial was to take place in Liberia and targets ‘healthy adults’ in the region – a category that includes but is not limited to those at highest risk. The study is likely to be moved to Guinea due to the containment of the infection in Liberia [32].

Like *Ebola ça Suffit,* the STRIVE trial was designed to avoid the use of placebo: it used an immediate and deferred vaccination strategy: ‘immediate’ vaccination was at enrollment, while ‘deferred’ was about six months after enrollment (significantly later than the ‘ring’ trial). Randomization was at individual rather than cluster level, and vaccine efficacy was assessed by the different rates of EVD infection observed between those vaccinated and those not yet vaccinated [30]. The merit of this study design from an ethical perspective is the avoidance of the use of a placebo in a study population at very high risk for an often fatal infection. However, the study was not blinded thereby introducing some bias: those who receive delayed vaccination may practice personal protection better than those who receive immediate vaccination. The length of the delay for those in the deferred group is an ethical concern: the incubation period of EVD is 21 days, and waiting 6 months for vaccination during an epidemic greatly increases the likelihood of exposure and infection. Also, the undue delay prolongs the study period during an epidemic that is known to decline over time even with limited intervention.

The design of the PREVAIL study also raises ethical issues: it has a placebo arm. While there was no evidence that either of the candidate vaccines offered any benefit over a placebo at the time of study design, there has been considerable discussion that the dire circumstances of the Ebola outbreak necessitated a different approach to studying the efficacy of candidate vaccines: access to experimental interventions should have higher priority over the use of a randomized clinical trial design for EVD vaccine efficacy studies [14, 16, 17].

We expect that with the evidence from the *Ebola çb Suffit* study trial, there will be reconsiderations on the delayed vaccination strategy and/or use of placebo in the STRIVE and PREVAIL as well as other EVD vaccine trials. It is our opinion that with the results from the *Ebola çb Suffit* study, it is difficult to satisfy the clinical equipoise principle. A placebo arm is ethically justified when there is genuine uncertainty in the scientific community as to which of several options is better [33]. It would thus be unethical to randomize people to delayed vaccination and/or placebo arms considering the high risk of infection and case fatality rates related to EVD. Likely savings from changes in the designs would ensure that maximum resources are committed to the candidate most likely to result in an effective implementation EVD prevention programs, including consideration of whether there are surrogate immunological markers of vaccine efficacy that could be used to assess subsequent vaccines.

### Ethical concerns with prioritizing healthcare providers for EVD vaccine research

One of the key ethical advantages of the ‘ring’ vaccination approach is that access to vaccination is directly related to risk – known exposure to an index case. We contend that in the circumstances of the West African Ebola outbreak, using known risk as a determinant for access is preferable to using occupational status. While experimental EVD vaccine research is associated with unknown risk, prioritization of healthcare workers as study participants for EVD vaccine research would also concentrate the benefits of research participation in a small population pool. Several authors have made the converse argument with respect to experimental interventions - that in the foreseeable event that access to experimental interventions must be rationed, healthcare workers should be prioritized over other candidates [34, 35].

Elsewhere, we have argued against prioritizing access to experimental *treatment* interventions for frontline workers on the basis that it could exacerbate existing inequalities [36–39] and that all people with a life threatening illness have the same moral right to treatment. We however consider that there is stronger moral justification for prioritizing healthcare *providers* – professional healthcare workers, laboratory personnel, ambulance staff, and burial or surveillance team members - as study participants for experimental EVD candidate vaccines for the following reasons. First, healthcare providers are among those at highest risk for EVD infection. Second, while an experimental vaccine might not confer any protective benefit, healthcare providers may be better placed to understand the potential risks and benefits of unproven agents, given that many might possess better knowledge of clinical and research concepts that are key to getting informed consent to experimental interventions. These include understanding the concept of randomization and the fact that EVD vaccine trials does not connote proven efficacy of candidate vaccine. For those personnel who may lack access to medical or clinical education such as those involved in public health tasks like burial, being in a workplace where discussion about these concepts can occur is likely to enhance understanding of EVD vaccine trials, and it can reduce the chances of a vaccine trial being misunderstood as an Ebola therapy (i.e., therapeutic misconception). The plausibility of professional healthcare providers not being familiar with clinical trials concepts is real: many years of wars and conflicts in the region have resulted in little investment in clinical trials in the region when compared with Southern and Eastern Africa. EVD vaccine researchers need to be aware of this potential challenge and develop contingency plans to improve healthcare providers’ understanding of clinical trial design and implementation either as trialists or as study participants.

Prioritizing healthcare providers as study participants for EVD vaccine clinical trials comes with other challenges. Targeting healthcare providers as study participants for EVD vaccine research has the potential to motivate governments of affected nations to *compel* health care providers to participate in vaccine research so they can provide care for patients infected with EVD. Compulsory recruitment of healthcare providers to participate in EVD response programmes has occurred in the past [40] and is a serious concern, as some health care providers hesitate to provide care because of their heightened risk for EVD [3, 41]. Yakubu et al. [39] argued that health care providers in EVD affected region are under no professional obligation to provide care for patients infected with EVD given that their safety in the occupational setting cannot be assured, even when following protocols regarding personal protective equipment (PPE).

Nevertheless we recognize that there are many professional healthcare providers who are willing to continue to provide care despite the risks. Prioritized access to experimental EVD vaccines should not, however, be promulgated as a benefit given that efficacy of an experimental intervention is uncertain. Members of the general population who are willing to provide health care services for patients with EVD infection, those who volunteer to serve as home based care providers, those who volunteer to provide treatment for patients with EVD at treatment centers, and those involved in public health practices such as contact tracing and public health education, should also be included as potential study participants, because they too are at heightened risk for EVD infection. As demonstrated by the ‘ring’ concept used in the rVSV-EBO trial in Guinea, whereby contacts and “geographic neighbors” [42] of an infected individual were identified and vaccinated, prioritizing access of all persons at risk for EVD infection reduces the risk of the trial being perceived as providing help to some and not to others [43]. Another strength of this model is that it uses the traditional public health process of contact tracing in an innovative way that is likely to strengthen contact tracing systems in-country; and it is likely to promote community support for contract tracing by demonstrating its potential benefit beyond identifying individuals not just for case isolation only. This model, we contend, is an excellent example of an approach that promotes equitable access of all persons at risk for EVD, and one that respects the principle of justice [44].

The altruism displayed by many healthcare workers who continue to provide care and support for patients with EVD should be a reason to strengthen health systems and safety nets in ways that reduces the risk for infection. This requires that a country’s disease surveillance systems be built or strengthened, more healthcare professionals trained, and laboratory capacity built and strengthened. Healthcare workers’ participation in an experimental EVD vaccine research would not (and should not) allay concerns about the effectiveness of current PPE [45], their heightened risk of infection [41] and poor access of infected healthcare workers to optimum care [46]. Experimental vaccination should also not be used to alleviate fear of infection. Therefore, due considerations need to be given to the standard of care provided for EVD vaccine trial participants [18].

Similar to Satalkar et al’s [34] observation, differential access to EVD care between international and local health care workers who become infected at work raises serious concerns. Differential access to care of persons infected with EVD during the current EVD outbreak has resulted in public discussions on the ethical obligation to respond to the health risk of local and international healthcare providersresponding to a national crisis [47]. These concerns are heightened if healthcare workers are engaged in research. Differential access to standard of care packages should not be dependent on nationality. Guaranteeing access to quality care for all study participants who contract EVD infection or when serious adverse events occur is important and would promote interest in EVD vaccine research [40]. Directly addressing access to medical care and management of adverse reactions associated with intervention research has been central in the demands community members make to researchers [48].

### Community engagement with EVD vaccine trial design and implementation

The sense of urgency surrounding EVD vaccine research and the need to conduct clinical trials before the current EVD epidemic ends, may compromise efforts to implement community education programs about EVD research. The benefits of community engagement extend beyond EVD trials. Over the long-term communities can become more literate in clinical science and be engaged in future research that directly impacts them. Moreover, lack of community education increases the risk of therapeutic misconception particularly in communities where research literacy is low [49]. Therapeutic misconception may lead to increased risky practices due to beliefs in vaccine efficacy. These challenges should not preclude the recruitment of non-health professionals for EVD clinical trials. It rather highlights why due considerations should be given to planning and implementing rigorous community education programs as part of the EVD vaccine research agenda.

It is important that communities located in research sites are made aware of research plans and the objectives for experimental vaccination. Community engagement is an ethical imperative as it ensures the integrity of research processes and the applicability of research outcomes. If we accept that proposed research should be relevant to the needs of the affected community, then the research design and the plans for implementation need to be deemed acceptable by the community hosting the research [50]. Community engagement facilitates the research process fitting into larger concerns regarding epidemiological landscapes, which is critical to understanding the EVD outbreaks as well as and making plans to prevent future outbreaks. A clinical trial that proceeds without established knowledge of the cultural nuances that affects perceptions about health, infection and vaccinations could potentially create negative unforeseen effects [43].

The community engagement process requires that community members are consulted at the beginning of the research project in an open, collaborative process [51]. Benefits of community consultation include strengthening local ownership of the research, effective communication between stakeholders, and the promotion of mutual understanding between trial communities and researchers [52]. Community engagement strategies include training of laypersons on ethics committees to reviewand provide constructive feedback on EVD vaccine protocols that addresses cultural context for study implementation, developing plans to engage and dialogue with formal and informal community structures about the research implementation [51], and discussing benefit-sharing mechanisms [34]. These processes require the use of multiple communication tools that can facilitate broad and wide-ranging communication procedureswithin the short time available to conduct this research. Such community engagement programs would help promote understanding about the differences between compassionate use of experimental EVD therapies and EVD vaccines research. It can also promote community support for recruiting and retaining study participants.

Low community involvement in EVD vaccine research design and implementation, coupled with poor coordination among stakeholders could cause community frustration, disillusionment and anger with the research process. This is because communities often want to engage in the agenda setting of medical research. These include questioning either the scientific logic that governs basic research, clinical experimentation, product development, clinical trial and product approval and the lengthy timescale required to complete it. These approaches are essential for promoting true community partnership in EVD vaccine research. The urgency surrounding EVD vaccine development must not preclude effective community engagement in research design and study implementations that are reconciled with the precariousness of the everyday life of study participants.

Health care providers are critical stakeholder to engage with during EVD vaccine clinical trial design and implementation. They need to be engaged in discussions about vaccine development as a matter of urgency, particularly regarding proposed plans to prioritize their access as study participants. Failure to actively engage healthcare providers in the study design and implementation may result in the slow recruitment of study participants due to myths, misconceptions, stigma and fear.

#### Addressing myths and misconceptions

Myths and misconceptions have been shown to affect the uptake of public health programs that involve vaccination [53]. Major concerns about vaccinations have had to do with the possible long-term consequences of harmful side effects and possible death. Spurious associations of immunization with morbidity and mortality [54, 55] and misconceptions that the vaccine itself may transmit a pathogen or cause sterility [56–58] have also caused concerns about vaccinations. Specific myths and misconceptions about EVD include beliefs that the West African governments invented the Ebola epidemic for the purpose of garnering foreign aid [23]. Another myth is that the disease was introduced to Africa by white foreigners for the purpose of conducting drug tests [59]. These myths were fueled largely by the mistrust of the government [22] and foreigners [23] following long, simultaneous histories of violent war and International Monetary Fund and World Bank led structural adjustment reforms that contributed to mass household poverty [60, 61] -- both were linked to illegal resource extraction by government and foreigners. Ironically, current EVD vaccine research are designed and mainly implemented by researchers from the North with collaborations that include government officials and donor partners thereby setting the stage for fueling myths and misconceptions about EVD vaccine research. Moreover, in some cases, health care providers are believed to transmit EVD and are widely stigmatized as a result [62, 63]. As such, mistrust of the research process may predispose health care workers to a new form of stigma. These myths and misconceptions could also affect uptake of proven vaccines.

It is therefore important to prevent and address myths and misconceptions about clinical trials in low research literacy communities through extensive and continued dialogue between researchers and community members. The objective is not only to promote community acceptance and support of proposed clinical trials but also to promote community literacy on the part of the researcher so as to ensure research designs are sensitive to and respects sociocultural norms and values. Overall, community engagement efforts should seek to ensure that the EVD vaccine trials are integrated with local measures that address differences in people’s capacities, preferences, cultural commitments, and socioeconomic and environmental circumstances. Then, EVD vaccine research efforts would be seen to be prioritizing and protecting the health of the affected communities [36, 37].

#### Negotiations for post-trial access

At the outset, clear and binding commitments are needed to ensure access to approved EVD vaccines as communities that participate in research and the countries that are affected by the epidemic may not be able to afford the final research products. The Hepatitis B vaccine is an example of a preventive agent that has not been affordable for a number of countries that were involved in its development [64]. Unfortunately, discussion about post-trial access to EVD vaccine is currently limited. Yet, post-trial access for communities that engage in international research is widely recognized as an important means to minimize exploitation and promote the social value of research [50]. Post-trial access to EVD vaccine is endorsed by the Declaration of Helsinki guideline 14, and the guideline 33 requires that arrangements for post-trial access be described in the research study protocol [65, 66]. Planning for post-trial access is therefore an ethical imperative.

However, as Zvonareva et al. [50] rightly pointed out, benefit sharing is complicated by the non-specificity regarding *who* ensures that benefits are made available. Folayan et al. [18] have proposed that the World Health Organization, which is currently engaged with coordinating the EVD response in the region, should be allocated the responsibility of ensuring post-trial access to EVD drugs and vaccines. The TRIPs Agreement of the World Trade Organization highly limits vaccine availability to low-income countries [67]. Unfortunately, specific discussions about EVD vaccine access and the impact of TRIPs have not been given priority attention [68]. The Global Alliance for Vaccine and Immunization (GAVI) had actively addressed some of the challenges associated with vaccine access in developing countries in the past and may likely work with pharmaceutical companies to address potential challenges that low-income countries may face [69]. While GAVI’s engagement with EVD vaccine access may be laudable, this approach promotes continued dependency of resource poor countries on donor support. One possible option to explore is differential pricings for EVD vaccines for countries that were engaged in the development of EVD vaccines [70, 71]. This would help make EVD vaccines affordable at least to these countries while in addition, price consideration is also given for other populations. Given that differential pricing may not entirely ensure easy access, another laudable option would be to, at the outset, engage effected West African governments in negotiations so that they can set firm prices that they affirm they can afford. Such legal discussions would ensure governments are held accountable to citizens in terms of their commitment to directly addressing future EVD outbreaks. This option further reduces donor dependency.

### Facilitating the translation of research outcomes to prompt product access

We need to learn and apply lessons from the recent past to EVD vaccine research and development. Poor engagement of community members in the design and implementation of research has marred past research programs [48], limited prompt translation of research outcomes to policies and programmes and the pace of community uptake and use of approved products. The poor uptake of polio vaccine in Northern Nigeria is a good example. Despite the known benefits associated with polio vaccination, the polio eradication program in Nigeria has been marred with challenges related to poor community trust and ownership of the polio vaccine program [72]. Patient access to any developed EVD vaccine is critical, and effective translation from clinical trials to implementation should be part of the planning process.

## Conclusion

We have argued that recruitment of study participants for experimental EVD vaccine research should not target only professional healthcare workers as this would entrench a sense of further marginalization in communities especially those where human right abuses are rampant. However, if the global agenda prioritizes enrollment of healthcare workers for experimental vaccines, it would be expedient for research teams to proactively hold public dialogues and discussions with community members and healthcare workers about the justification for prioritizing healthcare workers as study participants to experimental EVD vaccines, and how experimental EVD vaccine research differs from compassionate use of experimental therapies for EVD management.

We also advocate that it would be wise to consider the inclusion of other groups at heightened risk of infection alongside professional healthcare workers. We consider the ring design as a scientifically valid and ethically justified for the implementation of an EVD vaccine clinical trial during a major epidemic. The ring design ensures that all people at high risk of contracting EVD are recruited into the study equitably. The utilization of contact tracing as an element of the ring design helps facilitate the active engagement of communities with research implementation.

Public discussions about EVD vaccine trial design and implementation must be facilitated by trusted community members to engender trust, and members of communities in EVD stricken areas must have some say over how this prioritization is managed so that inequities are not further entrenched. The care needs of those involved in the vaccine trial who become EVD infected, as some inevitably will, must also form part of the discussion. Care must be of high standard, evidence based, and equitable for all research participants. The feasibility of future access to EVD vaccines in future outbreaks also needs to be included in ongoing discussions.

## Abbreviations

EVD: ebola virus disease
GAVI: global alliance for vaccine and immunization
HIV: human immunodeficiency syndrome
PrEP: pre exposure prophylaxis
TRIPS: trade related aspects of intellectual property
USA: Unites States of America
